# Dipolar addition to cyclic vinyl sulfones leading to dual conformation tricycles

**DOI:** 10.3762/bjoc.9.159

**Published:** 2013-07-15

**Authors:** Steven S Y Wong, Michael G Brant, Christopher Barr, Allen G Oliver, Jeremy E Wulff

**Affiliations:** 1 Department of Chemistry, University of Victoria, PO Box 3065 STN CSC, Victoria, BC, V8W 3V6, Canada; 2 Molecular Structure Facility, Department of Chemistry and Biochemistry, University of Notre Dame, 251 Nieuwland Science Hall, Notre Dame, IN, 46556, USA

**Keywords:** DFT calculations, dipolar addition, fluxional behavior, sulfones, VT NMR

## Abstract

Dipolar addition of cyclic azomethine imines with cyclic vinyl sulfones gave rise to functionalized tricycles that exhibited fluxional behavior in solution at room temperature. The scope of the synthetic methodology was explored, and the origin of the fluxional behavior was probed by NMR methods together with DFT calculations. This behavior was ultimately attributed to stereochemical inversion at one of two nitrogen centers embedded in the tricyclic framework. Two tetracycles were also synthesized, and the degree of signal-broadening in the NMR spectra was found to depend on the presence of substitution next to the inverting nitrogen center.

## Introduction

The 1,3-dipolar cycloaddition [1–3] represents a powerful methodology for the expedient regio- and stereospecific synthesis of five-membered ring N-, O- or S-containing heterocycles [4–5]. Various 1,3-dipoles can be used, including nitrones [6], azomethine ylides [7], diazoalkanes [8–9] and many others [10–12]. α,β-Unsaturated carbonyl compounds are often found to be good coupling partners for these cycloadditions, and in many cases the reaction rate can be enhanced through the addition of a Lewis acid that can coordinate to the carbonyl function, thereby lowering the energy of the olefin LUMO. By contrast, vinyl sulfones (with the exception of simple aryl vinyl sulfone species [13–21]) have been less-frequently employed as acceptors in 1,3-dipolar cycloadditions [22–25]. Indeed, we are only aware of a single prior example of a cyclic alkyl vinyl sulfone participating in a dipolar addition with an azomethine ylide [26]. In part, this lack of reactivity is due to a poor orbital overlap between the sulfone and olefin functions [27–29]; while vinyl sulfones are electrophilic (due to the inductive effects of the sulfone group), their LUMO cannot be as easily perturbed by the addition of a Lewis acid.

**Scheme 1:**
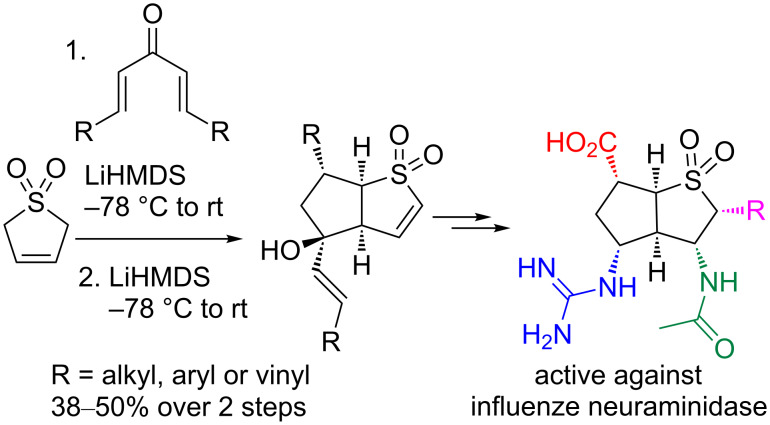
Synthesis of a conformationally constrained bicyclic sulfone, and application as an inhibitor of an enzyme target.

We previously described the synthesis of a family of [3.3.0] bicyclic vinyl sulfones starting from 3-sulfolene [30], and their elaboration to inhibitors of viral neuraminidase (Scheme 1) [31]. Understanding the conformational preferences of the underlying bicyclic structure was crucial to the design and assembly of such inhibitors. In keeping with our interest in the synthesis and utilization of conformationally constrained polycyclic sulfones, we sought to explore the cycloaddition between 2-sulfolene (**1a**) [32] and 1,3-dipoles (**2**, Table 1, see below) derived from 3-pyrazolidinone [33]. Reaction of **2** with acetylenic sulfones has been reported [34], but cyclic alkyl vinyl sulfones have not previously been demonstrated as coupling partners for **2**.

## Results and Discussion

A brief survey of reaction conditions revealed that dipole **2b** (easily prepared by condensing 3-pyrazolidinone with benzaldehyde) [33] could be reacted with **1a** in DMSO under reflux. When the two reagents were combined in equimolar quantities (Table 1, entry 2), the desired coupling product **3b** was isolated in 38% yield. Dipole **2** is known to be capable of homo-dimerization [35], and examination of the crude NMR spectra for the reaction revealed the presence of this dimer along with unreacted **1a**. We were gratified to find that simply increasing the amount of dipole to 3 equiv amplified the yield to 86%.

**Table 1:** Effect of dipole equivalents and electronics on the cycloaddition yield.

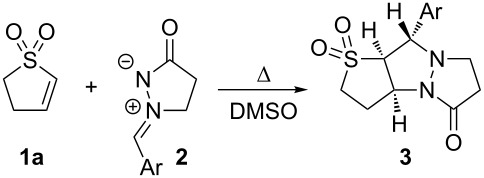			

entry	dipole **2**	1.0 equiv of **2** product (yield)	3.0 equiv of **2** product (yield)

1	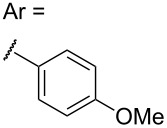 **2a**	**3a** (52%)	**3a** (84%)
2	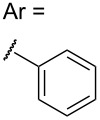 **2b**	**3b** (38%)	**3b** (86%)
3	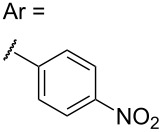 **2c**	**3c** (15%)	**3c** (56%)

Seeking to better understand how electronic properties in the dipole affected the reaction outcome, we repeated these coupling reactions using both electron rich (**2a**, Table 1, entry 1) and electron poor (**2c**, Table 1, entry 3) dipoles. When 1 equiv of dipole was used, a clear trend emerged wherein greater electron density was associated with an increased yield of product. At first glance this might seem counterintuitive, since more electron density at the hydrazone function should disfavor formation of one of the new C–C bonds in **3**. However, we speculate that the increase in electron density serves to disfavor dimerization *more* than 1,3-dipolar addition to **1a**. As a result, more dipole remains in solution to undergo the desired coupling. The effect of this change was somewhat muted when 3 equiv of dipole were used, resulting in synthetically useful yields of all three tricycles.

Characterization of the products **3a**–**3c** was complicated by the presence of very broad signals in both the ^1^H and ^13^C NMR spectra. Interestingly, however, HSQC analysis revealed that these broadened signals did not correlate with one another (i.e., one of the very broad ^1^H signals correlated to a sharp ^13^C signal, while one of the broad ^13^C signals correlated to a sharp signal in the ^1^H NMR spectrum). These observations suggested that signal broadening was not due to quadrupolar effects from the inclusion of the two nitrogen atoms (in which case broadening would be expected to be localized to certain regions of the molecular structure) but was more likely to be due to conformational switching of the molecule on a timescale similar to that of the NMR experiment.

**Figure 1:**
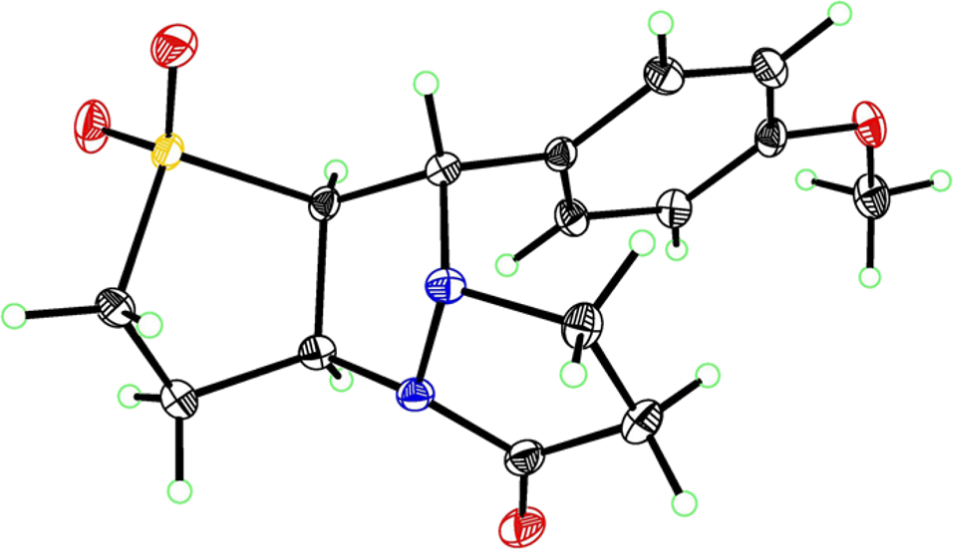
X-ray structure of **3a**.

We therefore turned to variable temperature NMR spectroscopy, both to assist in confirming the structures of the products, and to elucidate the conformational changes that were occurring. Extensive 1D and 2D NMR analysis of **3a** and **3b** at 353 K in DMSO-*d*_6_ (where sharp signals were observed for both compounds) confirmed the regio- and stereochemical outcome of the cycloaddition as that indicated for structure **3**. This structural analysis was subsequently confirmed by X-ray crystallographic analysis of **3a** (Figure 1) [36].

**Figure 2:**
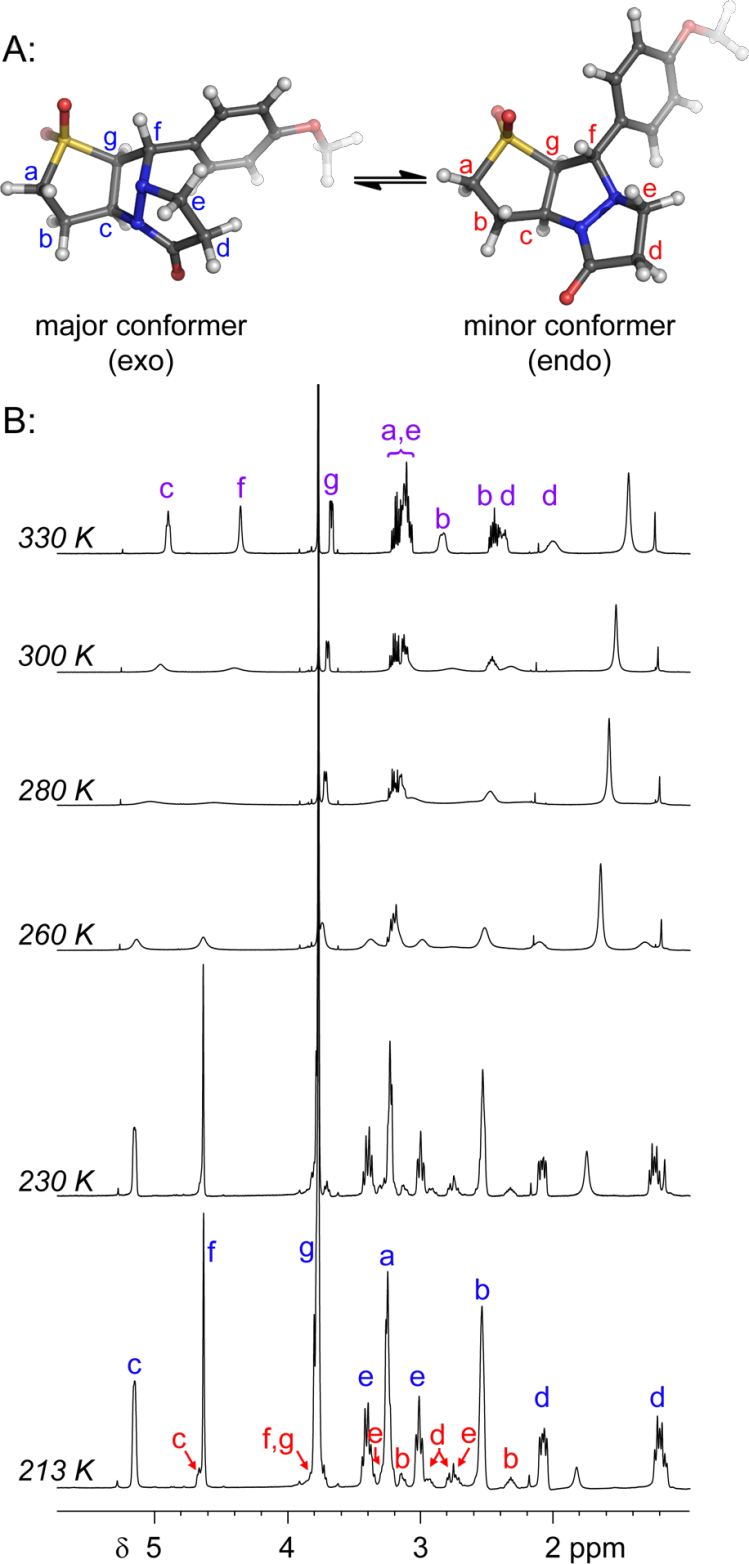
Assignment of major and minor conformations of **3a**; **A:** DFT-calculated conformers; **B:** Collected ^1^H NMR spectra at various temperatures, in CDCl_3_. Assignments in Figure 2B refer to the structures in Figure 2A, where blue = major isomer, red = minor isomer and purple = equilibrating mixture.

Further VT NMR studies of **3a** in CDCl_3_ (Figure 2B) revealed a substantial broadening of several key ^1^H NMR signals below 330 K, with a coalescence temperature of approximately 280 K. At lower temperatures, two distinct conformations could be identified; by 213 K these were sufficiently well-resolved to permit integration (revealing a ≈7:1 ratio of the two conformers) and more extensive NMR analysis (HSQC, HMBC, ROESY, TOCSY and EXSY).

The most significant difference between the NMR data for the two conformations was the substantial upfield shift for the two protons adjacent to the carbonyl (‘d’ in Figure 2) in the major conformer. This was consistent with this methylene group being substantially shielded by the electron-rich aromatic ring, as would be expected for the conformation observed in the X-ray structure (Figure 1). This upfield shift was not present in the minor conformer, suggesting that the pyrazolidinone ring had moved away from the aromatic ring’s magnetic influence.

Analysis of several lines of evidence (including nOe information from the NMR studies, construction of likely molecular models, and comparison with known fluxional behavior for related structures [37]) strongly suggested that these differences could best be explained by the tricycle “breathing” through stereochemical inversion at the nitrogen atom distal to the carbonyl. To test this hypothesis, we carried out DFT calculations [38] on structures in which the pyrazolidinone and sulfolane rings were oriented *syn* or *anti* to one another (i.e., “endo” or “exo” conformations, respectively). Geometry optimization resulted in the two lowest-energy structures shown in Figure 2A. Compellingly, the difference in Δ*H*_f_ between these two structures (0.93 kcal/mol in favor of the exo conformer) agrees very well with the experimental difference in energy calculated from NMR integration of the two conformations at 213 K (0.8 kcal/mol).

The calculated structure of the lower-energy exo conformer was similar to the solid-state structure shown in Figure 1, and was consistent with the nOe and NMR shielding data discussed above. Seeking further evidence linking the calculated exo and endo structures to the observed major and minor conformers, we calculated the ^13^C NMR shifts for our geometry-optimized structures, and compared these to the shifts obtained experimentally for the two conformations (Table 2). These agreed exceptionally well for those carbons not connected to the sulfone (relativistic effects were not well handled by our computational method; shifts for carbons close to the sulfone could therefore not be accurately calculated).

**Table 2:** Comparison of selected ^13^C NMR data.

	δ (^13^C): major conformer (ppm)			δ (^13^C): minor conformer (ppm)		
^13^C	calculated	observed	Δ (δ)	calculated	observed	Δ (δ)

c	55.6	55.7	0.1	53.0	54.7	1.7
d	30.3	31.3	1.0	36.1	37.5	1.4
e	42.1	42.1	0.0	51.8	50.3	1.5
f	68.7	67.8	0.9	67.8	71.3	3.5

Of particular note is the effect of the nitrogen inversion on carbons “d” and “e”. In the exo conformer, these atoms are brought into proximity with the shielding aromatic ring, resulting in atypically upfield chemical shifts. This shielding effect is not present in the endo conformer, and so these ^13^C shifts return to more typical values. The difference is substantial: 6 ppm for carbon “d” and 8 ppm for carbon “e”. The excellent agreement between theory and experiment for these two carbons in both conformations, together with good qualitative agreement between calculated and observed ^1^H NMR shifts (not shown) and other data summarized above, allows us to unambiguously assign the major conformer as exo, and the minor conformer as endo.

We next sought to evaluate whether the method could be extended toward the preparation of the analogous tetracycles, through the use of bicyclic vinyl sulfones as substrates. To this end, we attempted to react dipoles **2a** and **2b** with the readily prepared [39] bicycle **1b** (Table 3, entry 1). Unfortunately, no cycloaddition adducts were observed even when additional heating was employed, either thermally in a sealed tube or in a microwave reactor.

**Table 3:** Cycloadditions with bicyclic sulfones, and related control experiments.^a^

entry	sulfone **1**	dipole	product (yield)

1	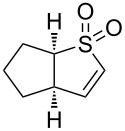 **1b**	**2a** or **2b**	–

2	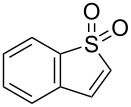 **1c**	**2a**	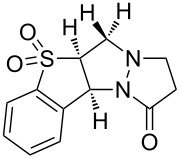 **4** (37%)

3	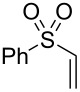 **1d**	**2a**	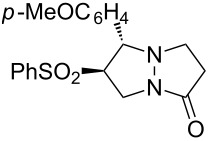 **5** (68%)

4	**1c**	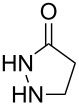 **6**	**4** (67%)

5^b^	**1c**	**6**	**4** (70%)

6^c^	**1c**	**2b**	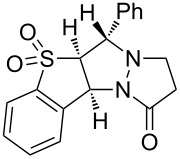 **7** (95%)

^a^3 equiv of **2** or **6** were used for each experiment. ^b^25 equiv of paraformaldehyde were added. ^c^Reaction was carried out in bromobenzene under reflux.

Bicycle **1b** is somewhat more hindered than **1a**, which is presumably the reason behind its lack of reactivity. In an attempt to attenuate the steric hindrance, we next examined the reaction of **2a** with bicycle **1c** (Table 3, entry 2). We were pleased to observe a tetracyclic product in this case (albeit in modest yield) but were surprised to find that the anisole ring was not included in the isolated compound. Indeed, adduct **4** appeared to result from transiminization of dipole **2a** with formaldehyde (generated by the thermal decomposition of DMSO [40]), followed by selective cycloaddition of the more reactive dipole species. This suggests that **2a** may be too hindered to react easily with bicycle **1c**, but that this hindrance can be mitigated by in situ exchange to generate a dipole lacking the aromatic ring. Compound **4** displayed sharp signals in the ^1^H and ^13^C NMR spectra, presumably due to the lack of an aromatic substituent next to the sp^3^-hybridized nitrogen atom. Electronically, aryl vinyl sulfone **1c** is unlike the alkyl sulfones **1a** and **1b**. To confirm that its aberrant reactivity was not due to electronic factors, we carried out the cycloadditon of **2a** with the simpler acyclic aryl vinyl sulfone **1d** (Table 3, entry 3). As anticipated, this reaction proceeded smoothly to afford the expected product **5** in reasonable yield. To confirm that the anomalous methylene group in product **4** originated from the decomposition of DMSO, we reacted bicycle **1c** with 3-pyrazolidinone (**6**). Once again we isolated tetracycle **4**, this time in a considerably improved yield of 67% (Table 3, entry 4). Adding an excess of paraformaldehyde to the reaction mixture (Table 3, entry 5) had no significant effect on the yield. Finally, to explore the dipolar addition in the absence of competing transiminization processes, we reacted bicycle **1c** with 3 equivalents of **2b**, in refluxing bromobenzene (Table 3, entry 6). This provided an excellent yield of the desired tetracycle **7**, confirming that even hindered dipolar additions of this type can proceed if no alternative pathways are open to the reactants. The additional aromatic substituent in **7** (relative to **4**) once again contributed to significant broadening in both the ^1^H and ^13^C NMR spectra.

## Conclusion

Our results comprise the first syntheses of tricyclic sulfones by 1,3-dipolar cycloadditions of azomethine imines with cyclic alkyl vinyl sulfones. Only single diastereomers were obtained in each case, and the yields were respectable when an excess of the dipole was used. Both electron-donating and electron-withdrawing substituents on the dipole’s aromatic function were tolerated. Initial attempts to synthesize a tetracyclic analogue resulted in the appearance of compound **4**, resulting from an unexpected transimination/cycloaddition sequence. By avoiding the transimination pathway, the original target tetracycle **7** could be isolated.

Of potentially greater interest than the syntheses themselves, tricycles **3a**–**3c** (as well as tetracycle **7**) exhibited an unusual fluxional behavior at room temperature, which was ultimately traced to stereochemical inversion at the sp^3^-hybridized nitrogen. This behavior is consistent with previous examples of fluxional alkaloids, but represents a new structural type that may find application in supramolecular or medicinal chemistry. DFT- and NMR-based studies agreed remarkably well in implicating the two interconverting species as the “endo” and “exo” conformers shown in Figure 2.

## Experimental

**General methods.** Unless otherwise stated, all reagents were purchased from commercial suppliers and used without further purification. DMSO was dried by distillation over CaH_2_. ^1^H chemical shifts are reported in parts per million (ppm, δ scale) downfield from tetramethylsilane, and are referenced to residual protium in the NMR solvent (CDCl_3_: δ 7.26). Likewise, ^13^C chemical shifts are referenced to the carbon resonances of the solvent (CDCl_3_: δ 77.00). TLC plates were visualized by exposure to KMnO_4_ stain. Accurate masses were obtained using an orbitrap MS. Infrared spectra were collected using an FT IR spectrometer.

**General procedure for the dipolar cycloaddition.** The vinyl sulfone (0.5 mmol) and dipole (3 equiv) were combined in dry DMSO (2.0 mL), and the resulting solution was heated under reflux in a sand bath. After 24 h, the solution was concentrated under vacuum at 50 °C. The remaining black viscous oil was purified by flash-column chromatography using a 2–10% gradient of methanol in dichloromethane, to afford the desired adduct as a brown solid.

**Tricycle 3a:** mp 189–192 °C; IR (film, cm^−1^): 1694, 1514, 1302, 1254, 1112; ^1^H NMR (CDCl_3_, 300 MHz, rt) δ 7.17–7.07 (m, 2H), 6.92 (d, *J* = 8.8 Hz, 2H), 5.01 (br s, 1H), 4.46 (br s, 1H), 3.81 (s, 3H), 3.75 (dd, *J* = 7.8, 1.9 Hz, 1H), 3.34–3.08 (m, 4H), 2.80 (br s, 1H), 2.59–2.27 (m, 2H), 1.80 (br s, 1H); ^13^C NMR (CDCl_3_, 75 MHz, rt) δ 160.3 (C), 129.7 (CH), 125.9 (C), 114.7 (CH), 70.4 (br CH), 69.0 (br CH), 55.3 (CH), 55.2 (CH_3_), 48.2 (CH_2_), 44.6 (br, CH_2_), 32.8 (br, CH_2_), 25.8 (br, CH_2_); HRMS–ESI (*m/z*): calcd for [C_15_H_18_N_2_O_4_S]^+^, 322.0987; found, 322.0983.

**Tricycle 3b:** mp 134–137 °C; IR (film, cm^−1^): 1693, 1303, 1114; ^1^H NMR (CDCl_3_, 300 MHz, rt) δ 7.43–7.37 (m, 3H), 7.27–7.20 (m, 2H), 5.00 (br s, 1H), 4.46 (br s, 1H), 3.79 (dd, *J* = 7.8, 2.6 Hz, 1H), 3.33–3.12 (m, 4H), 2.87 (br s, 1H), 2.58–2.34 (m, 2H), 2.01 (br s, 1H); ^13^C NMR (CDCl_3_, 75 MHz, rt) δ 134.3 (C), 129.2 (CH), 129.2 (CH), 128.3 (CH), 70.5 (CH), 69.3 (CH), 55.2 (CH), 48.1 (CH_2_), 44.9 (br, CH_2_), 33.4 (br, CH_2_), 25.4 (br, CH_2_); HRMS–ESI (*m/z*): calcd for [C_14_H_16_N_2_O_3_S]^+^, 292.0882; found, 292.0883.

**Tricycle 3c:** mp 201–203 °C; IR (film, cm^−1^): 1694, 1520, 1349, 1305, 1113; ^1^H NMR (CDCl_3_, 300 MHz, rt) δ 8.27 (d, *J* = 8.8 Hz, 2H), 7.61 (d, *J* = 8.6 Hz, 2H), 4.81 (br t, *J* = 6.4 Hz, 1H), 4.32 (br d, *J* = 4.9 Hz, 1H), 3.69 (dd, *J* = 7.8, 5.8 Hz, 1H), 3.40–2.94 (m, 5H), 2.77–2.36 (m, 3H); ^13^C NMR (CDCl_3_, 75 MHz, rt) δ 148.4 (C), 142.4 (C), 128.9 (CH), 124.4 (CH), 69.6 (CH), 55.3 (CH), 47.9 (CH_2_); HRMS–ESI (*m/z*): calcd for [C_14_H_15_N_3_O_5_S]^+^, 337.0732; found, 337.0730.

**Tetracycle 4:** oil; IR (film, cm^−1^): 1713, 1305, 1149; ^1^H NMR (CDCl_3_, 300 MHz, rt) δ 7.79–7.56 (m, 4H), 5.89 (d, *J* = 8.1 Hz, 1H), 4.17 (t, *J* = 7.6 Hz, 1H), 3.94 (d, *J* = 10.4 Hz, 1H), 3.62 (dt, *J* = 12.2, 9.2 Hz, 1H), 3.19 (ddd, *J* = 12.2, 9.2, 4.6 Hz, 1H), 2.87 (dd, *J* = 10.2, 7.7 Hz, 1H), 2.78 (dt, *J* = 17.8, 9.1 Hz, 1H) 2.66 (ddd, *J* = 17.8, 9.4, 4.6 Hz, 1H); ^13^C NMR (CDCl_3_, 75 MHz) δ 175.7 (C), 139.4 (C), 134.7 (C), 134.4 (CH), 130.9 (CH), 128.0 (CH), 121.0 (CH), 64.1 (CH), 56.6 (CH), 55.3 (CH_2_), 46.1 (CH_2_), 30.1 (CH_2_); HRMS–ESI (*m/z*): calcd for [C_12_H_12_N_2_O_3_S + H]^+^, 265.0642; found 265.0640.

**Bicycle 5:** mp 184–186 °C; IR (film, cm^−1^): 1708, 1515, 1307, 1251, 1149; ^1^H NMR (CDCl_3_, 300 MHz, rt) δ 7.80, (d, *J* = 7.3 Hz, 2H), 7.60 (t, *J* = 7.4 Hz, 1H), 7.48 (t, *J* = 7.6 Hz, 2H), 7.25 (d, *J* = 8.7 Hz, 2H), 6.81 (d, *J* = 8.7 Hz, 2H), 4.35 (dd, *J* = 12.8, 3.7 Hz, 1H), 4.05–3.95 (m, 2H), 3.79 (s, 3H), 3.54 (dd, *J* = 12.6, 8.0 Hz, 1H), 3.47 (dt, *J* = 11.9, 9.1 Hz, 1H), 3.10 (ddd, *J* = 12.0, 9.5, 4.7 Hz, 1H), 2.80 (dt, *J* = 17.5, 8.9 Hz, 1H), 2.61 (ddd, *J* = 17.5, 9.3, 4.8 Hz, 1H); ^13^C NMR (CDCl_3_, 75 MHz, rt) δ 174.2 (C), 160.1 (C), 137.5 (C), 134.2 (CH), 129.4 (CH), 128.6 (CH), 127.3 (C), 114.3 (CH), 72.0 (CH), 68.5 (CH), 55.3 (CH_3_), 45.3 (CH_2_), 42.6 (CH_2_), 30.0 (CH_2_); HRMS–ESI (*m/z*): calcd for [C_19_H_20_N_2_O_4_S + H]^+^, 373.1217; found 373.1214.

**Tetracycle 7:** oil; IR (film, cm^−1^): 1698, 1304, 1150; ^1^H NMR (CDCl_3_, 300 MHz, rt) δ 7.96–7.84 (m, 1H), 7.81 (d, *J* = 7.7 Hz, 1H), 7.73 (td, *J* = 7.5, 1.2 Hz, 1H), 7.64 (br t, *J* = 7.7, 1H), 7.47–7.40 (m, 3H), 7.32–7.22 (m, 2H), 6.06 (d, *J* = 8.1 Hz, 1H), 4.78 (br s, 1H), 4.42 (dd, *J* = 8.2, 2.5 Hz, 1H), 3.34–3.17 (m, 1H), 3.15–3.02 (m, 1H), 2.36–2.18 (m, 1H), 1.94–1.62 (m, 1H); ^13^C NMR (CDCl_3_, 75 MHz) δ 172.4 (C), 138.9 (C), 134.5 (CH), 134.2 (C), 134.1 (C), 131.0 (CH), 129.4 (CH), 129.4 (CH), 128.9 (CH), 121.2 (CH), 70.1 (CH), 68.1 (CH), 56.1 (CH), 43.8 (CH_2_), 32.6 (CH_2_); HRMS–ESI (*m*/*z*): calcd for [C_18_H_16_N_2_O_3_S + Na]^+^, 363.0774; found 363.0771.

## Supporting Information

Copies of ^1^H, ^13^C and DEPT-135 spectra for all new compounds, VT-NMR data and 2D NMR spectra for **3a**, crystallographic data for **3a**, and absolute energies and Cartesian coordinates for calculated conformers.

File 1 Detailed measurement data.
