# Supplementary material for: Dipolar addition to cyclic vinyl sulfones leading to dual conformation tricycles
Source: Beilstein J Org Chem. 2013 Jul 15;9:1419–25. doi: 10.3762/bjoc.9.159 (PMC3740686; doi:10.3762/bjoc.9.159)
Supplement: File 1 — Detailed measurement data. [file Beilstein_J_Org_Chem-09-1419-s001.pdf]

Supporting Information

for

Dipolar addition to cyclic vinyl sulfones leading to dual conformation tricycles

Steven S. Y. Wong,[1] Michael G. Brant,[1] Christopher Barr,[1] Allen G. Oliver[2] and Jeremy E. Wulff*[1]

Address: [1]Department of Chemistry, University of Victoria, PO Box 3065 STN CSC, Victoria, BC, V8W 3V6, Canada and [2]Molecular Structure Facility, Department of Chemistry and Biochemistry, University of Notre Dame, 251 Nieuwland Science Hall, Notre Dame, IN, 46556, USA

Email: Jeremy E. Wulff* - wulff@uvic.ca.

* Corresponding author

Detailed measurement data

Index

1. ^1H, ^{13}C, and DEPT-135 NMR spectra for compound **3a** in $CDCl_3$ at rt (300/75 MHz) S2
2. ^1H NMR spectrum for compound **3a** in $CDCl_3$ at 328K (500 MHz) S3
3. ^1H NMR spectrum for compound **3a** in DMSO-d_6 at 353K (500 MHz) S3
4. COSY and HSQC NMR spectra for compound **3a** in DMSO-d_6 at 353K (500 MHz) S4
5. ^1H NMR spectra for compound **3a** in $CDCl_3$ from 213K to 330K (500 MHz) S5
6. COSY and HSQC NMR spectra for compound **3a** in $CDCl_3$ at 213K (500 MHz) S6
7. ROESY NMR spectra for compound **3a** in $CDCl_3$ at 213K (500 MHz) S7
8. ^1H, ^{13}C, and DEPT-135 NMR spectra for compound **3b** in $CDCl_3$ at rt (300/75 MHz) S8
9. ^1H, ^{13}C, and DEPT-135 NMR spectra for compound **3c** in $CDCl_3$ at rt (300/75 MHz) S9
10. ^1H, ^{13}C, and DEPT-135 NMR spectra for compound **4** in $CDCl_3$ at rt (300/75 MHz) S10
11. ^1H, ^{13}C, and DEPT-135 NMR spectra for compound **5** in $CDCl_3$ at rt (300/75 MHz) S11
12. ^1H, ^{13}C, and DEPT-135 NMR spectra for compound **7** in $CDCl_3$ at rt (300/75 MHz) S12
13. Geometry-optimized structure for **3a-**endo: Energy and Cartesian coordinates S13
14. Geometry-optimized structure for **3a-**exo: Energy and Cartesian coordinates S14

1. ^{1}H, ^{13}C, and DEPT-135 NMR spectra for compound **3a** in $CDCl_3$ at rt (300/75 MHz)

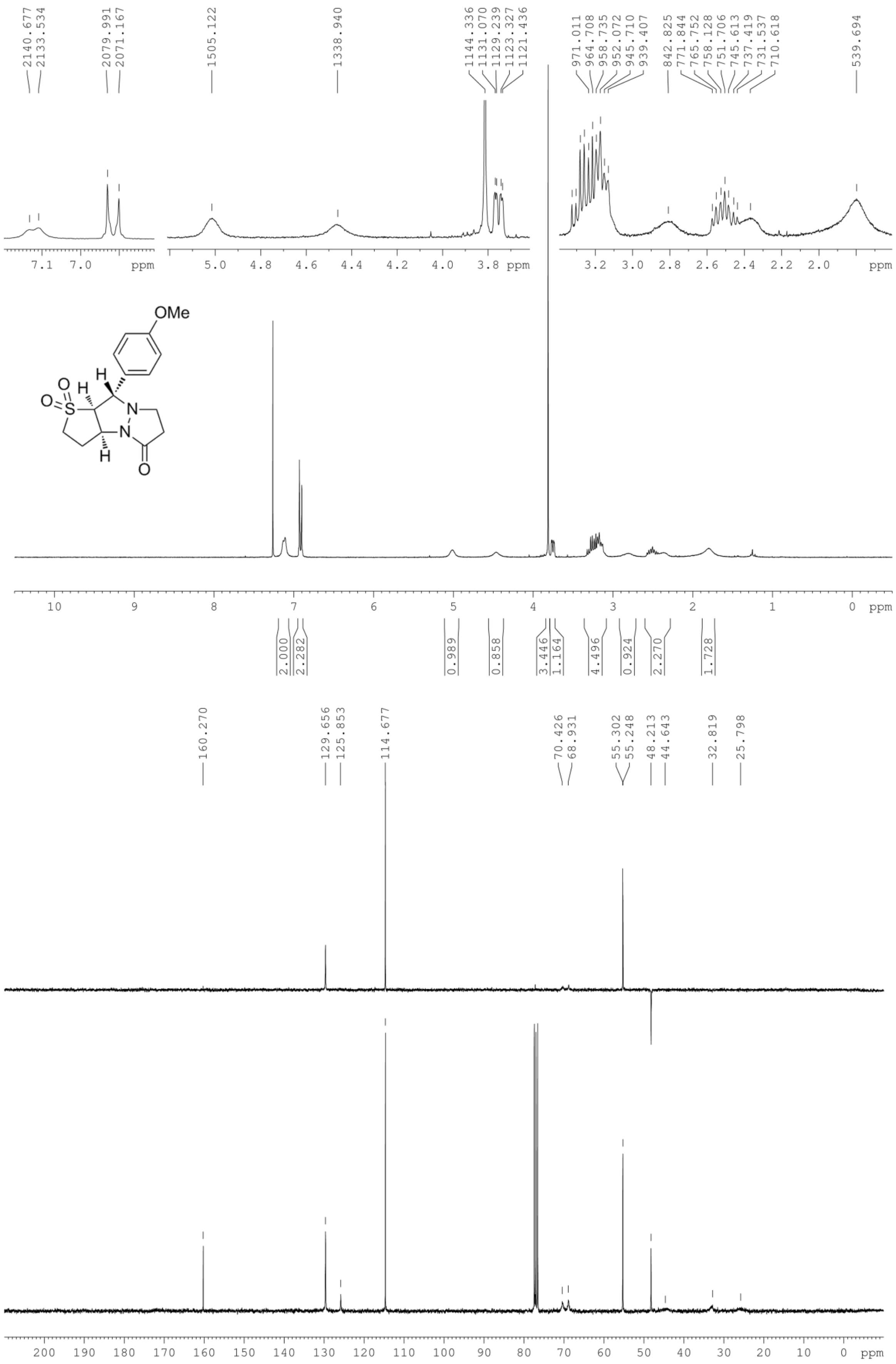

2. ^{1}H NMR spectrum for compound **3a** in $CDCl_3$ at 328K (500 MHz)

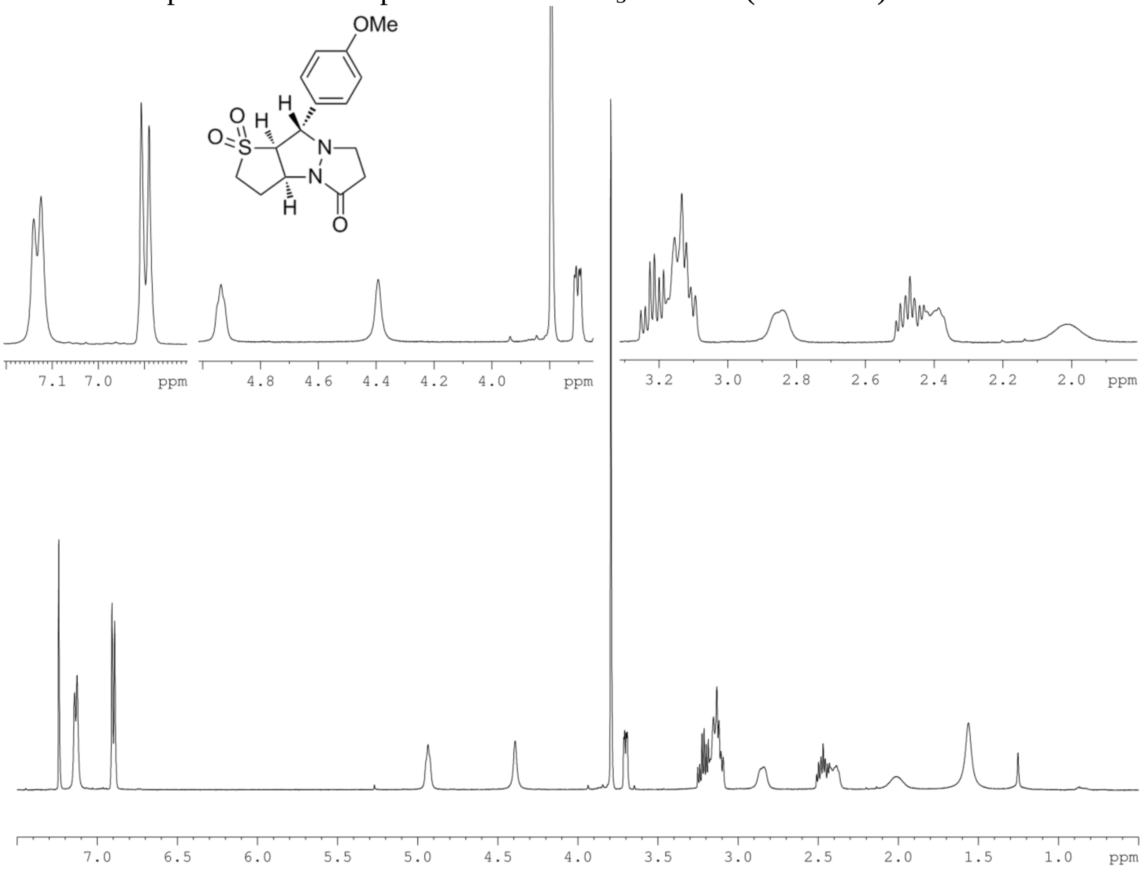

3. ^{1}H NMR Spectrum for compound **3a** in DMSO-d_6 at 353K (500 MHz)

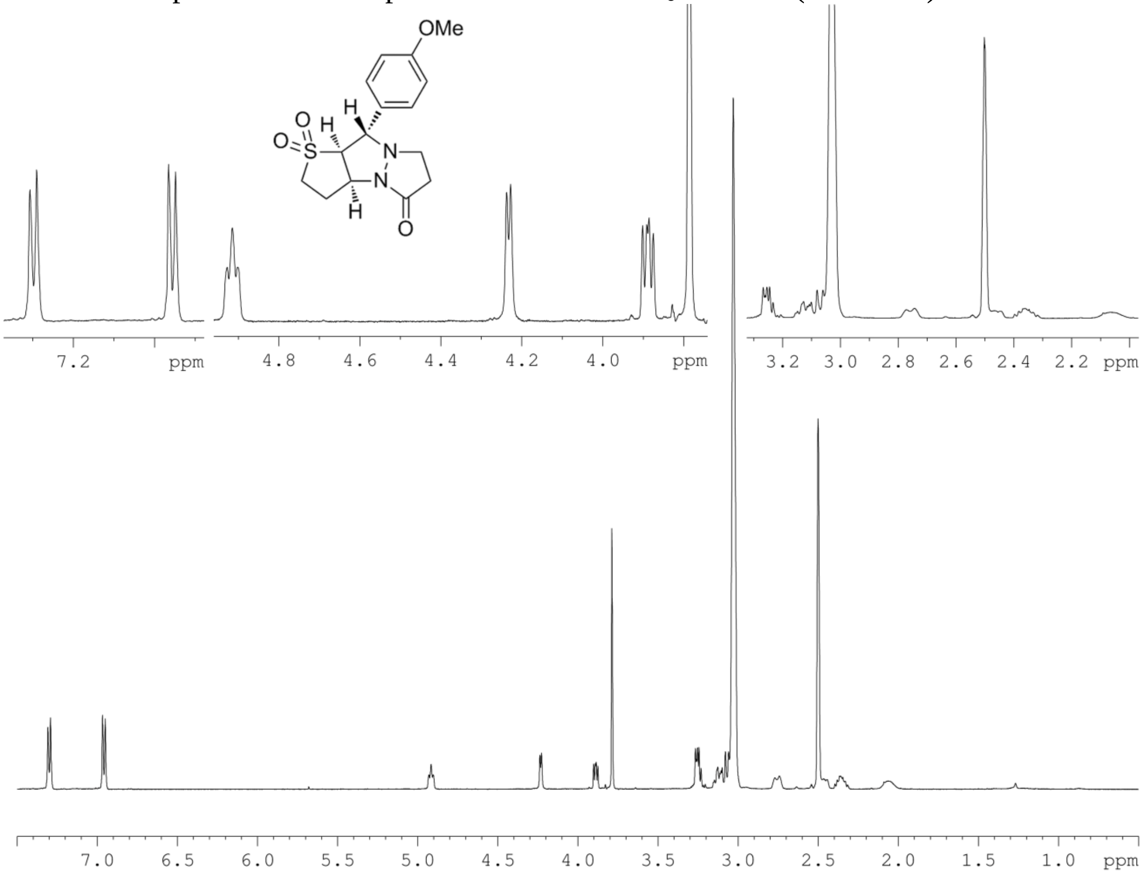

4. COSY and HSQC NMR spectra for compound **3a** in DMSO-d_6 at 353K (500 MHz)

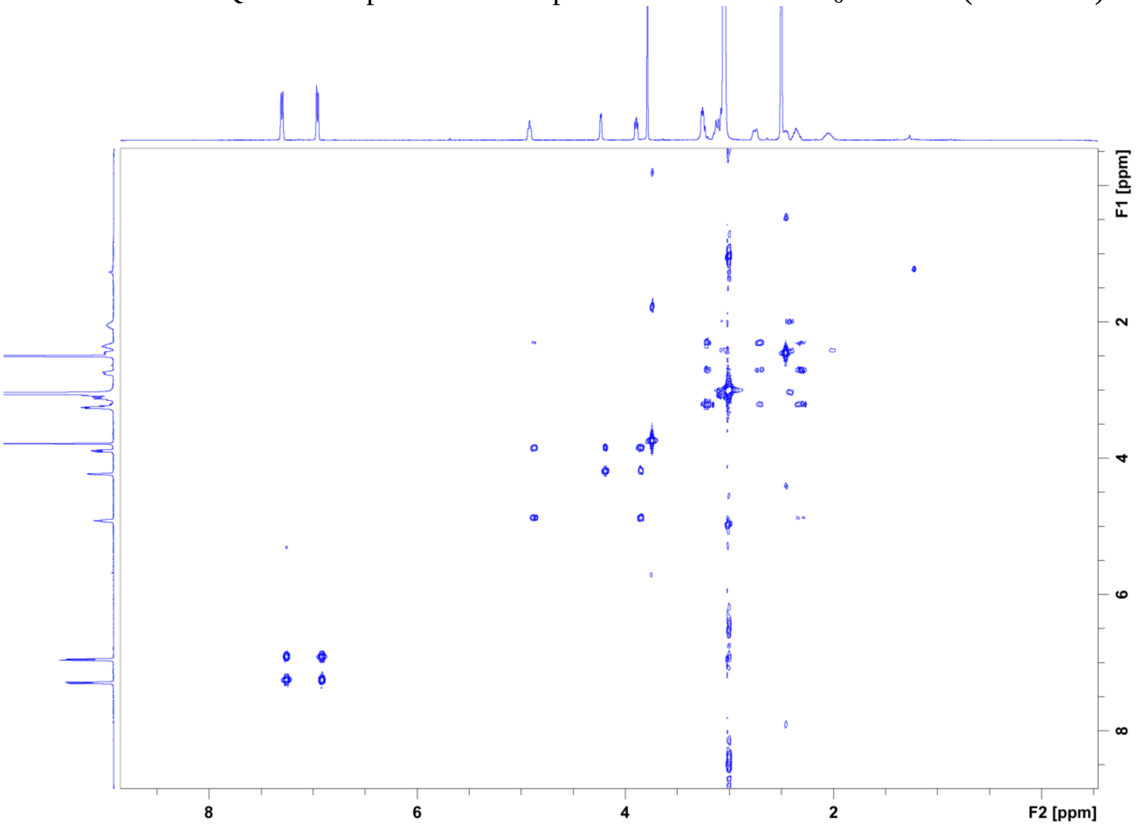

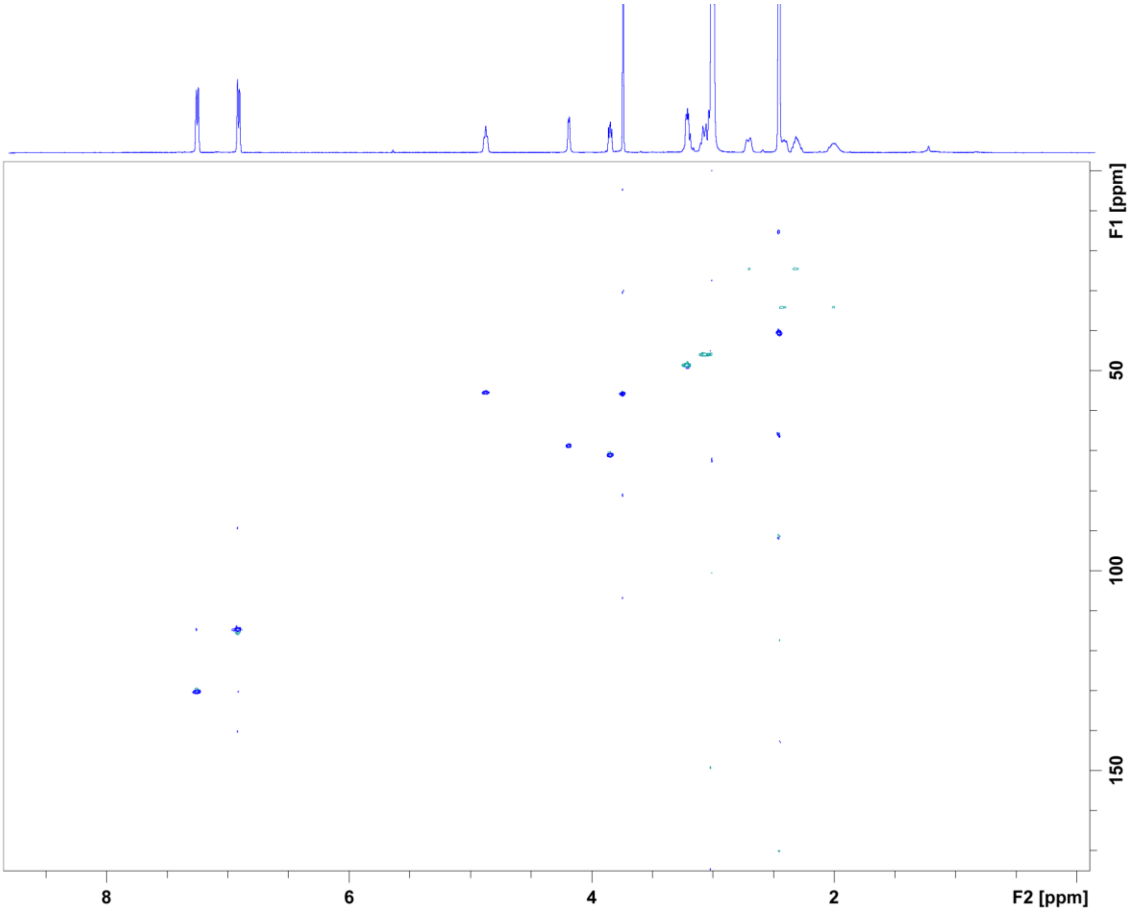

5. ^{1}H NMR spectra for compound **3a** in $CDCl_3$ from 213K to 330K (500 MHz)

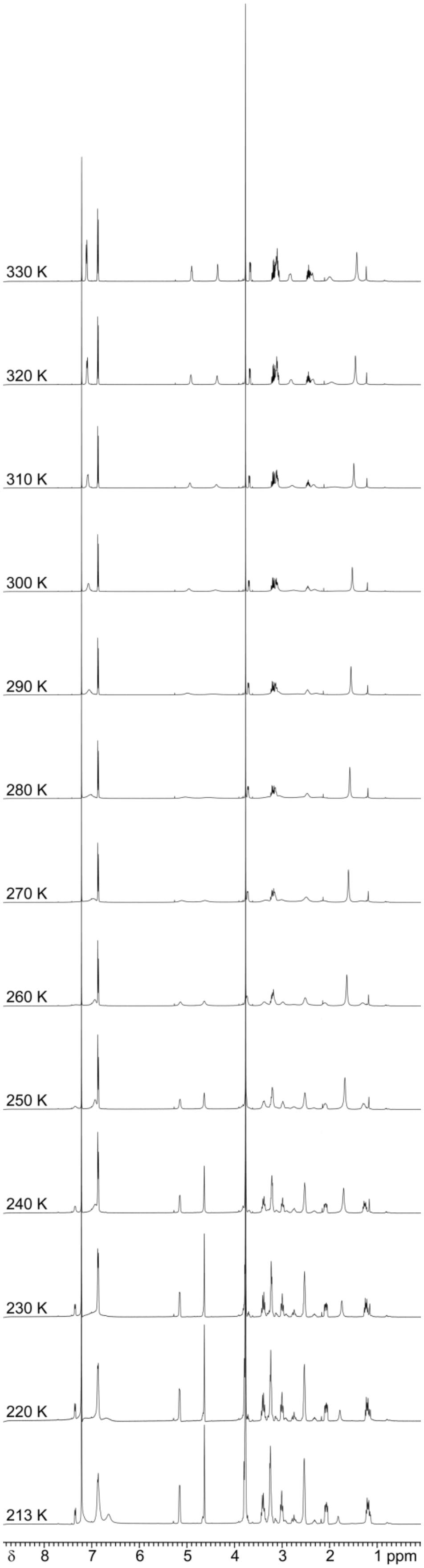

6. COSY and HSQC NMR spectra for compound **3a** in $CDCl_3$ at 213K (500 MHz)

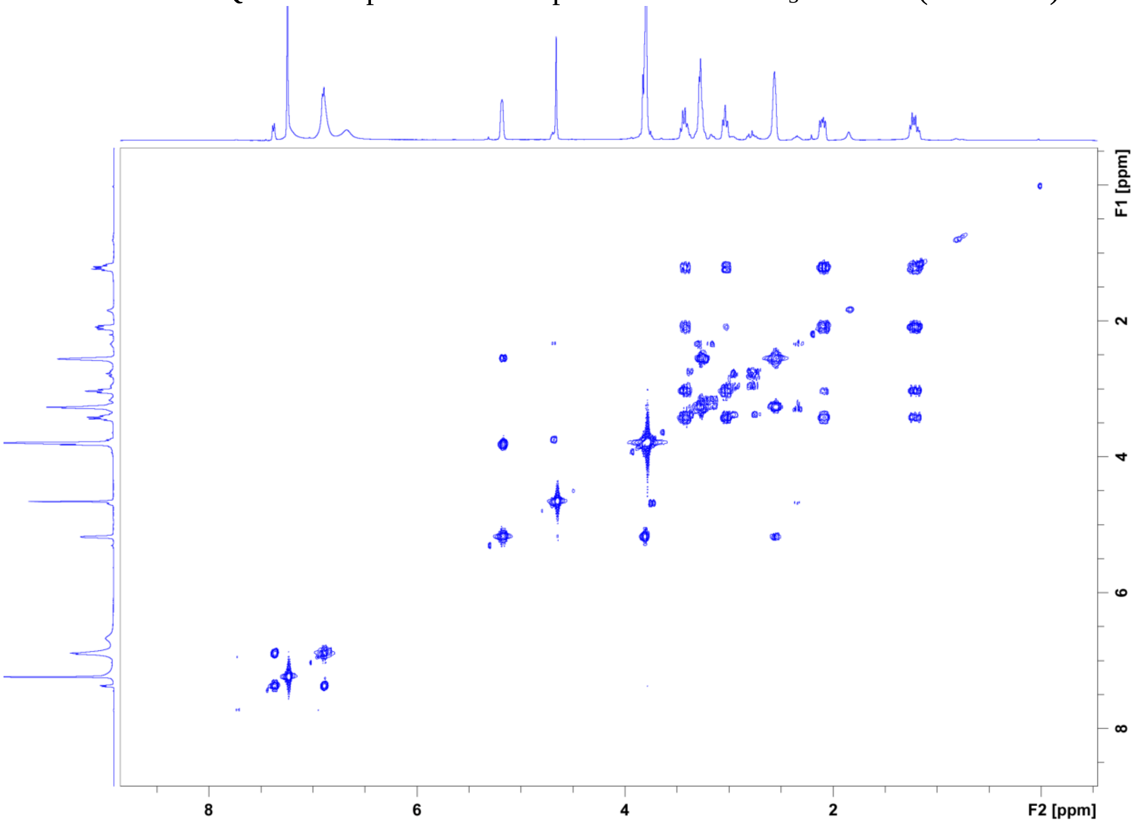

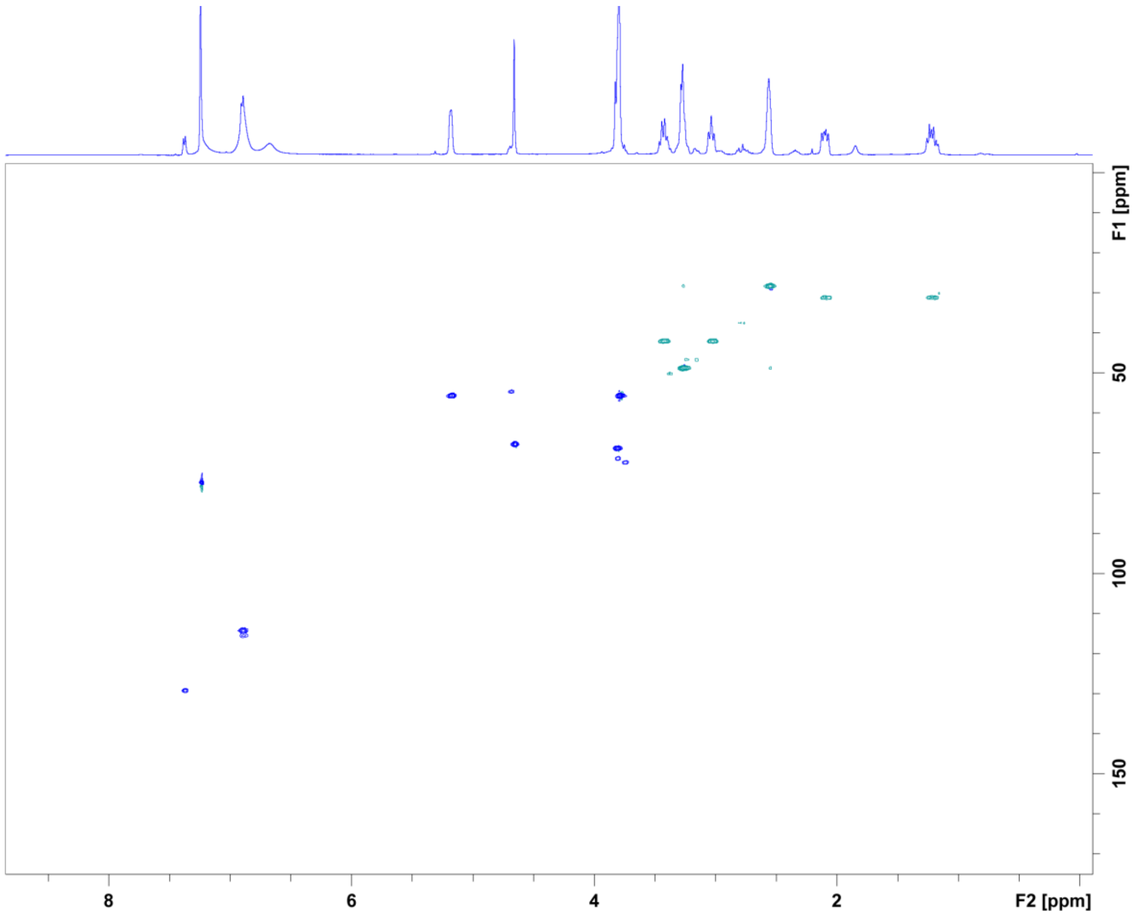

7. ROESY NMR spectra for compound **3a** in $CDCl_3$ at 213K (500 MHz)

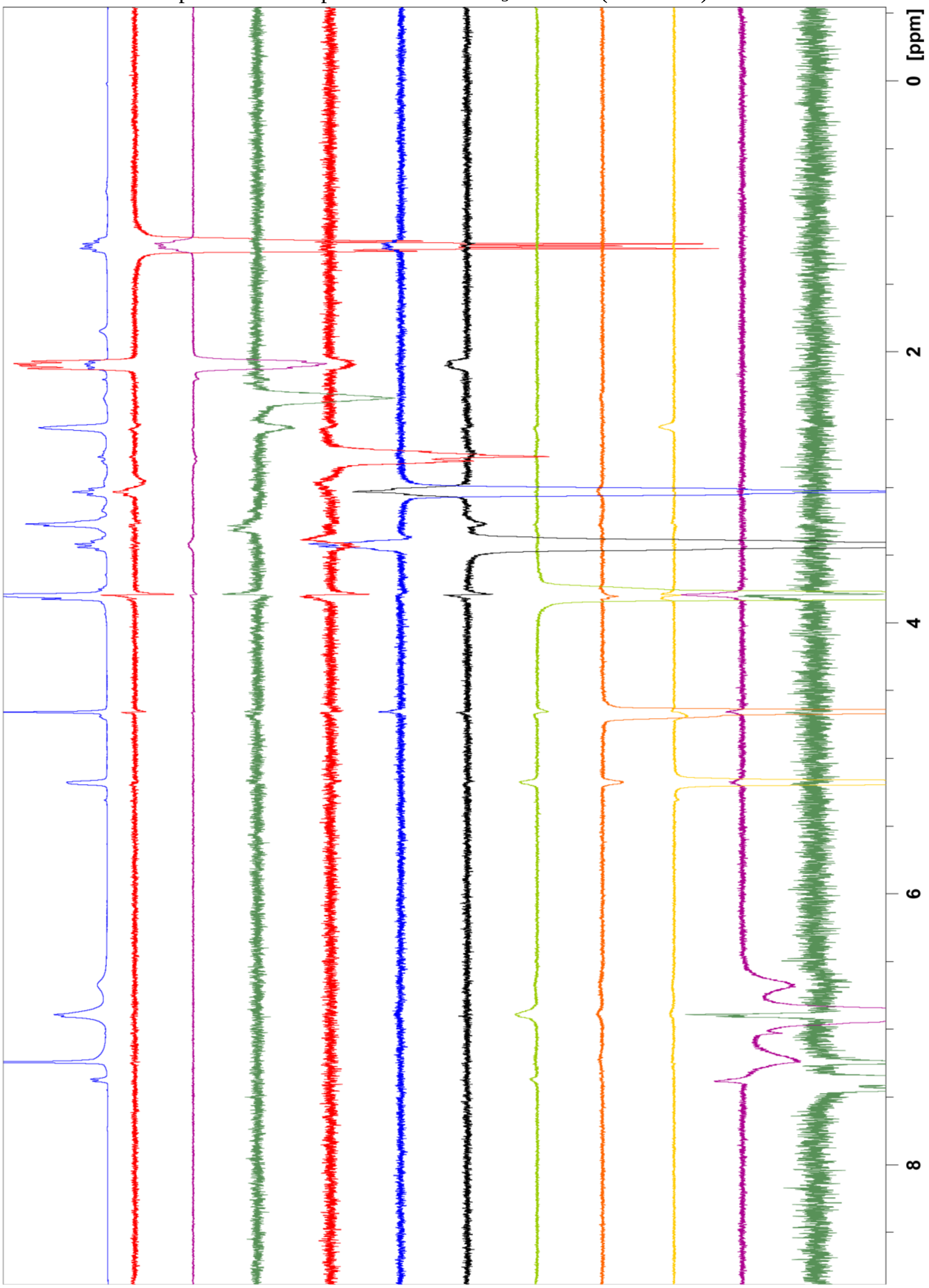

8. ^{1}H, ^{13}C, and DEPT-135 NMR spectra for compound **3b** in $CDCl_3$ at rt (300/75 MHz)

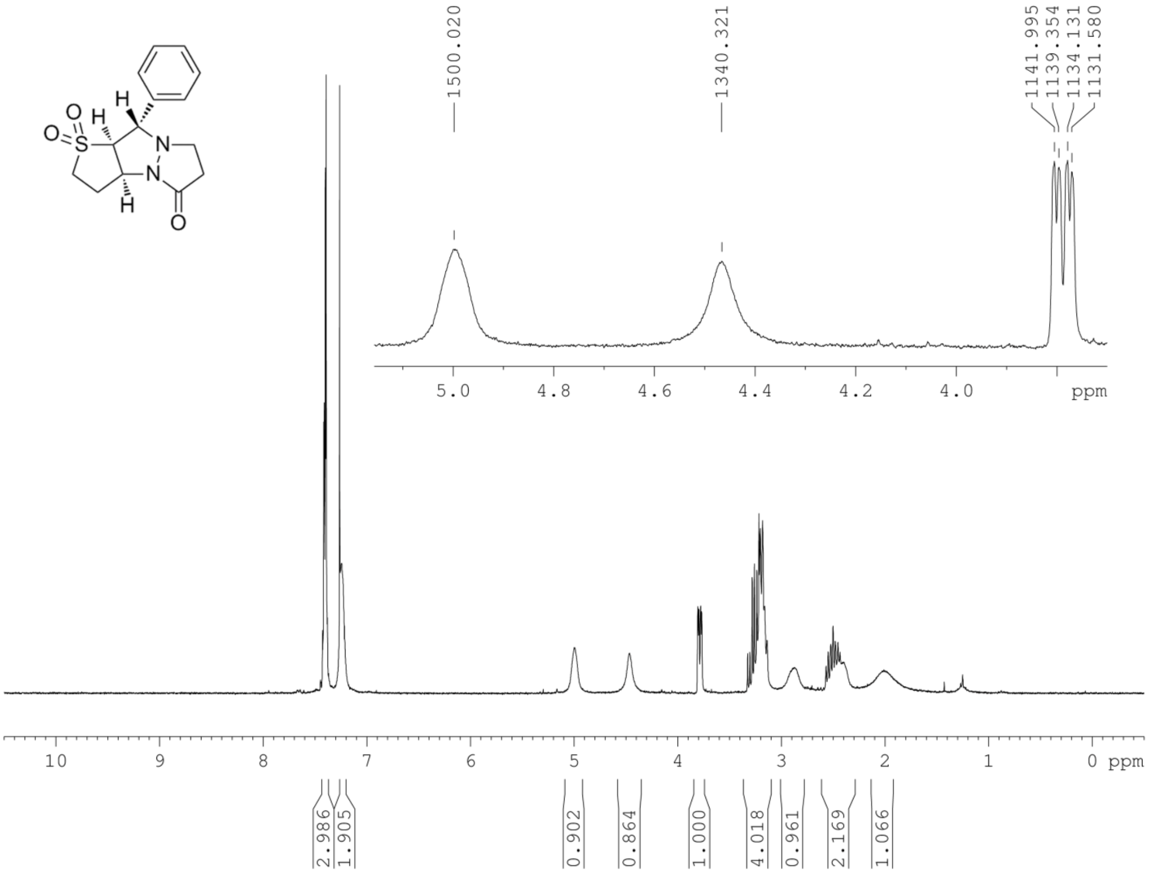

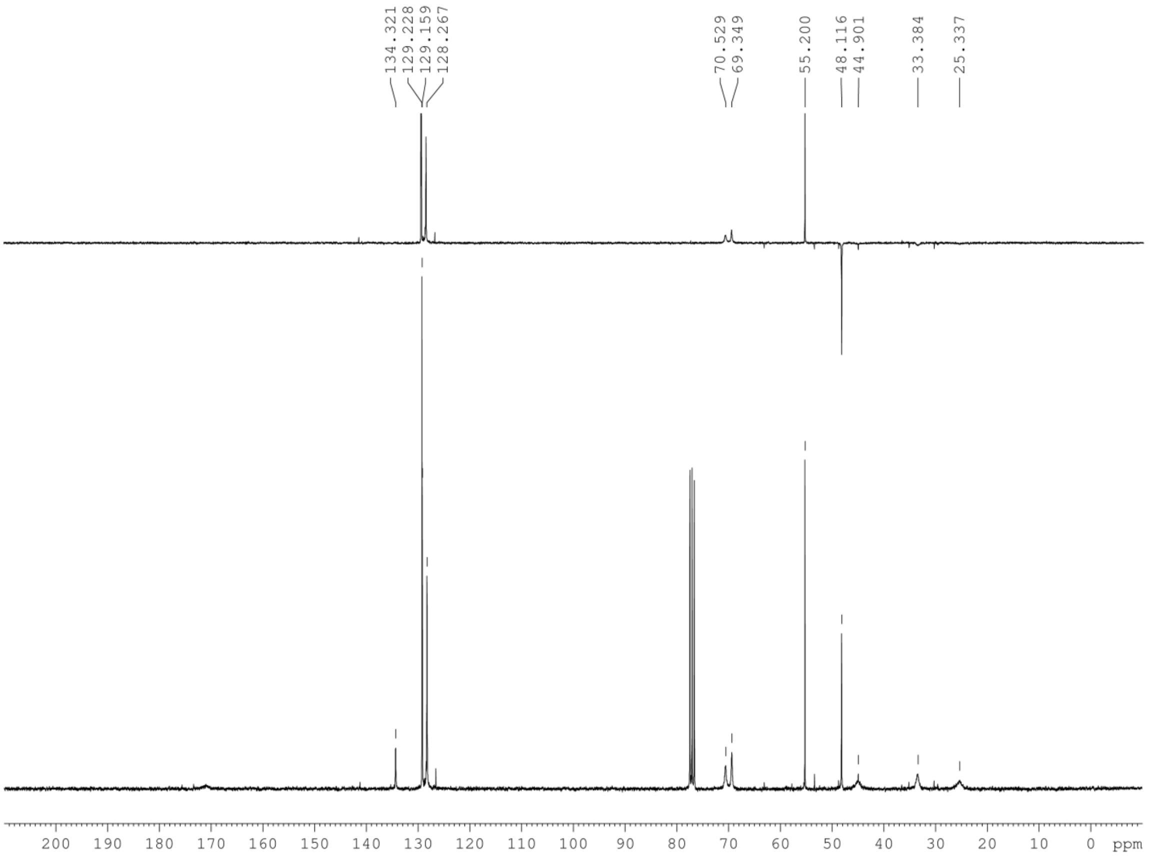

9. ^{1}H, ^{13}C, and DEPT-135 NMR spectra for compound **3c** in $CDCl_3$ at rt (300/75 MHz)

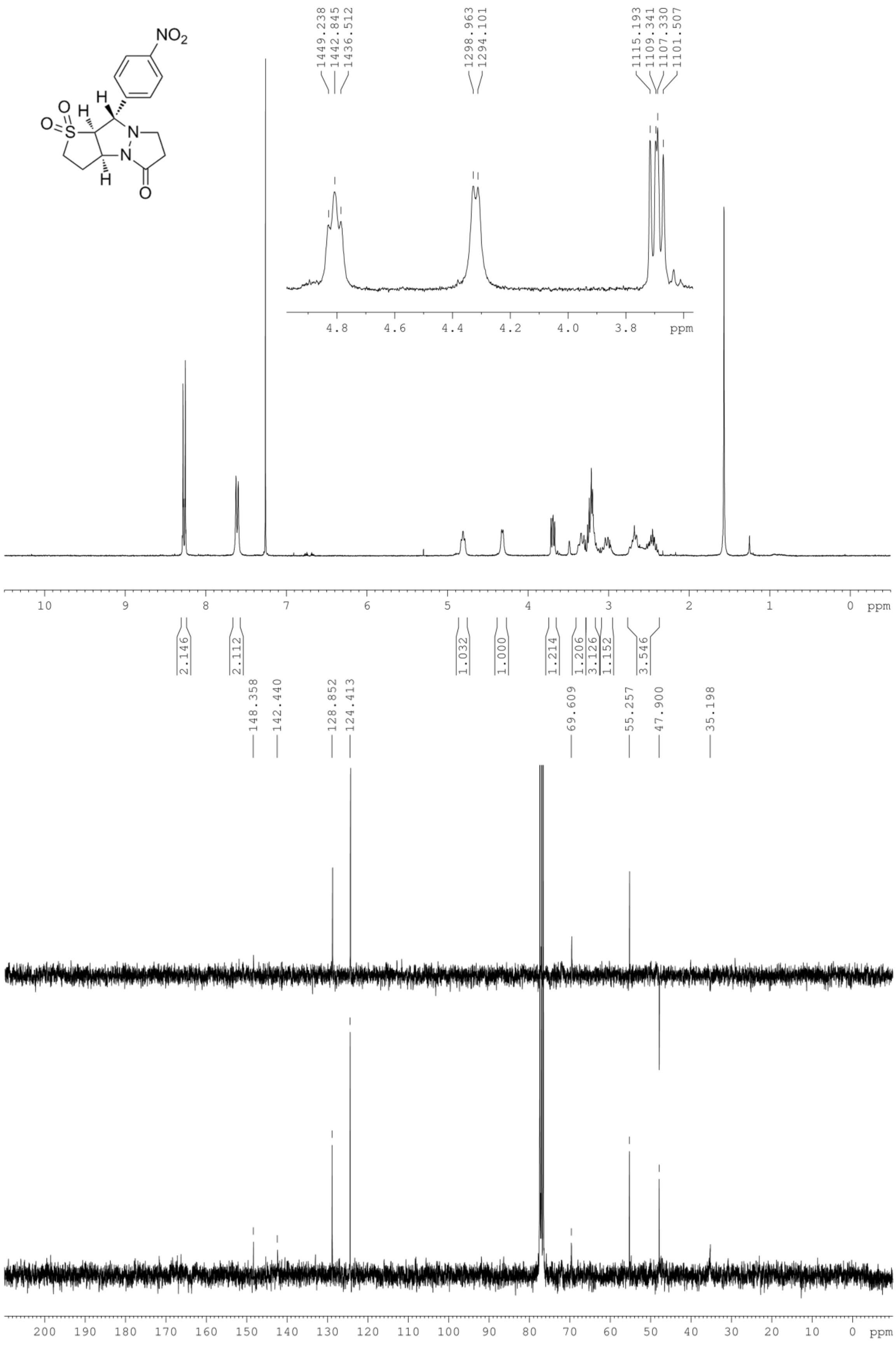

10. ^{1}H, ^{13}C, and DEPT-135 NMR spectra for compound **4** in $CDCl_3$ at rt (300/75 MHz)

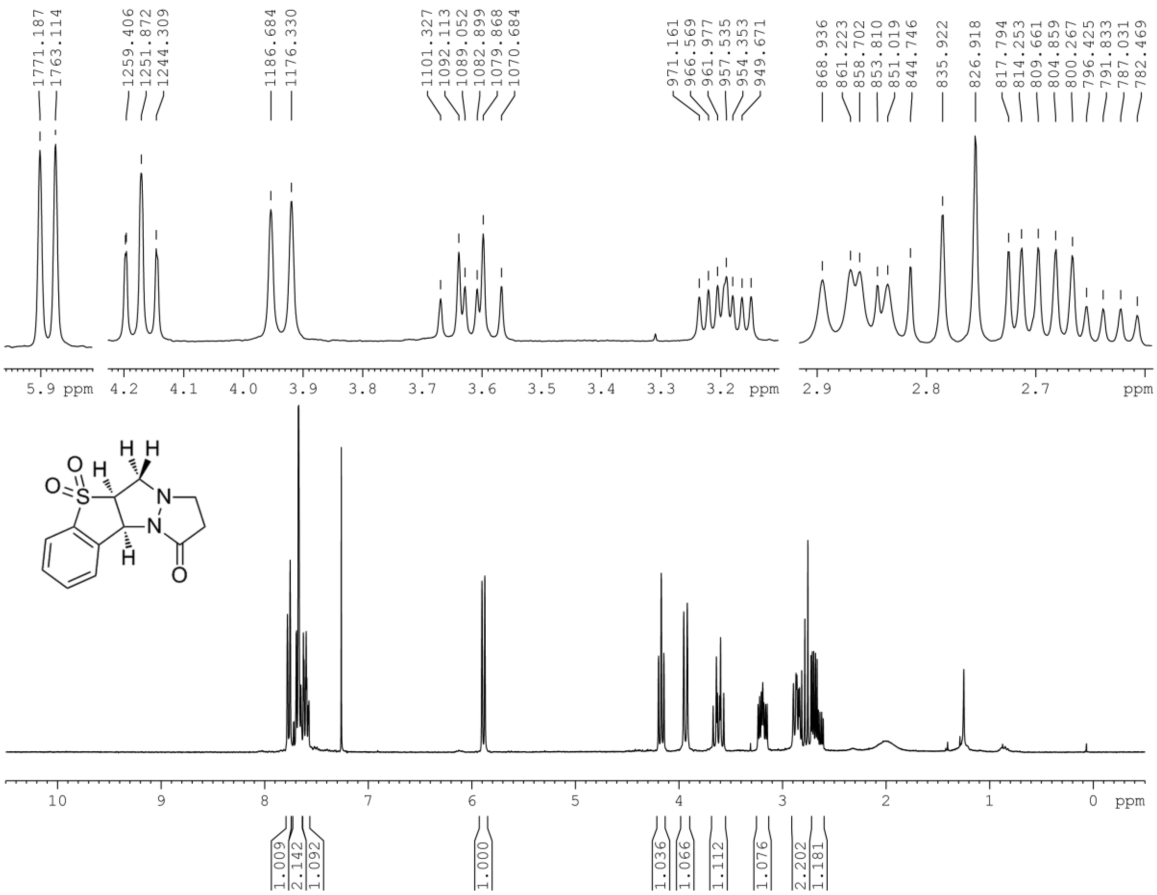

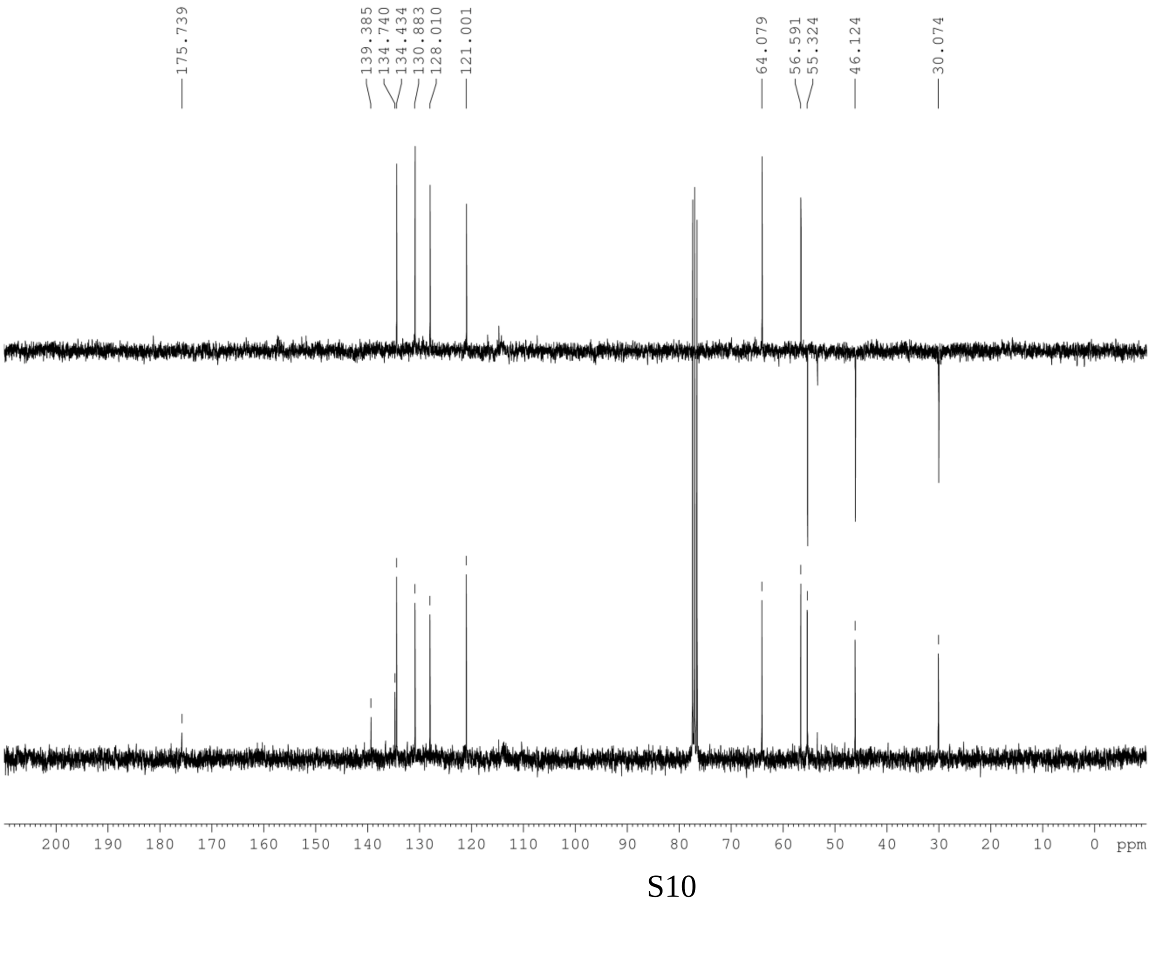

11. ^{1}H, ^{13}C, and DEPT-135 NMR spectra for compound **5** in $CDCl_3$ at rt (300/75 MHz)

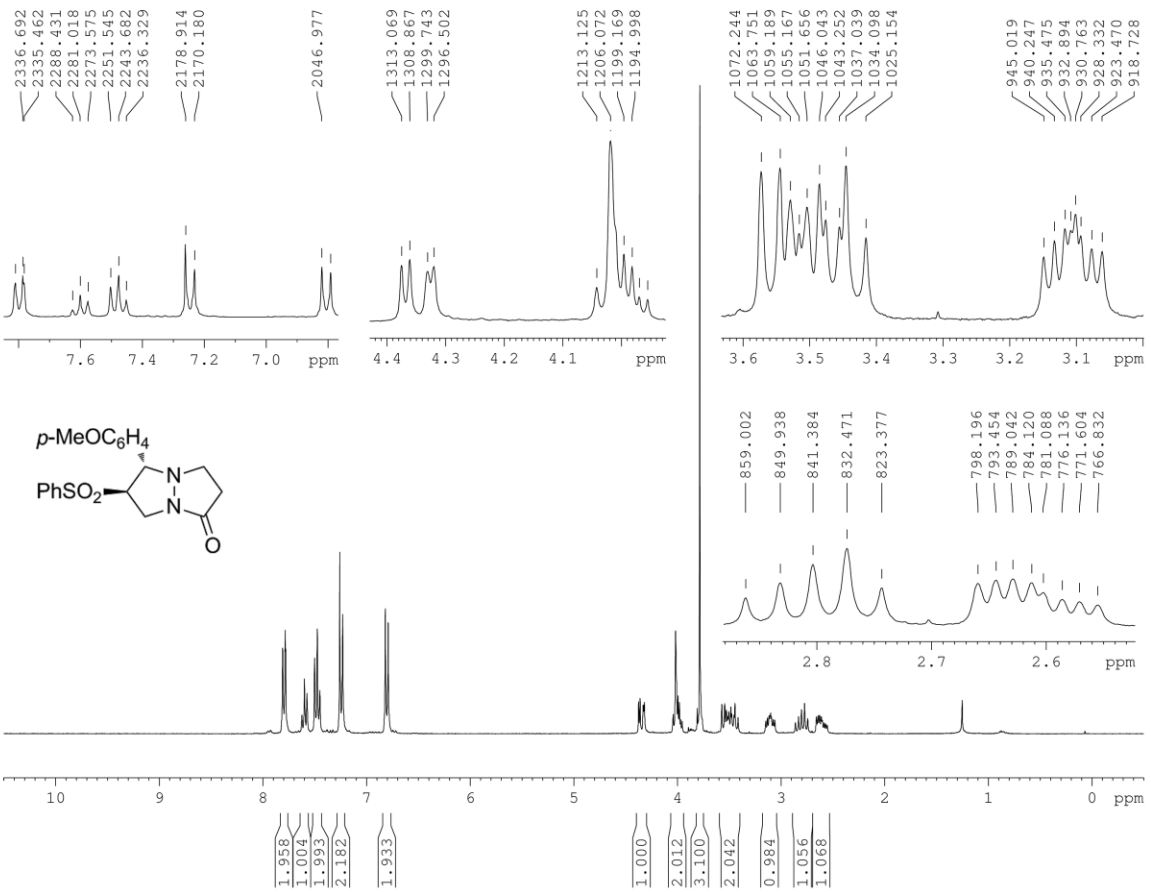

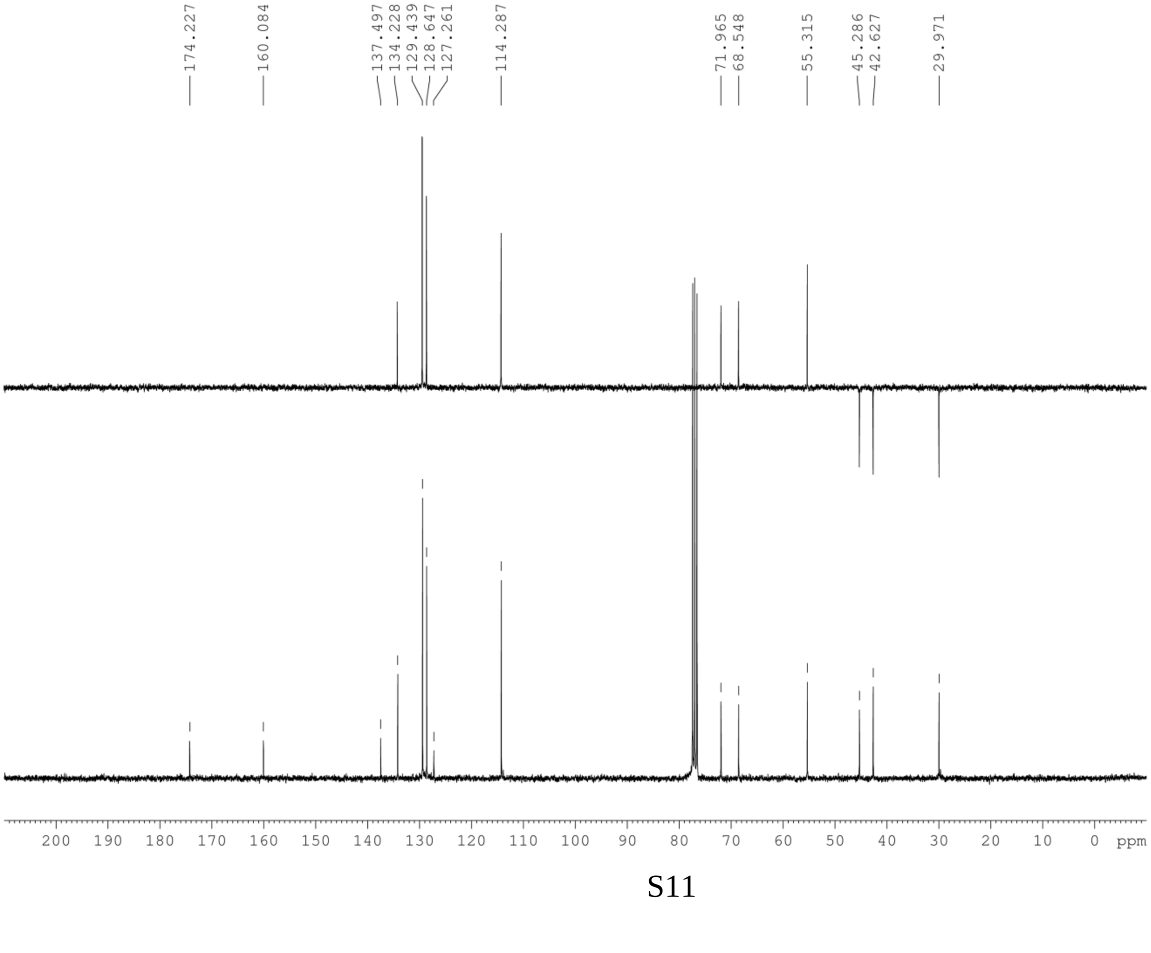

12. ^{1}H, ^{13}C, and DEPT-135 NMR spectra for compound **7** in $CDCl_3$ at rt (300/75 MHz)

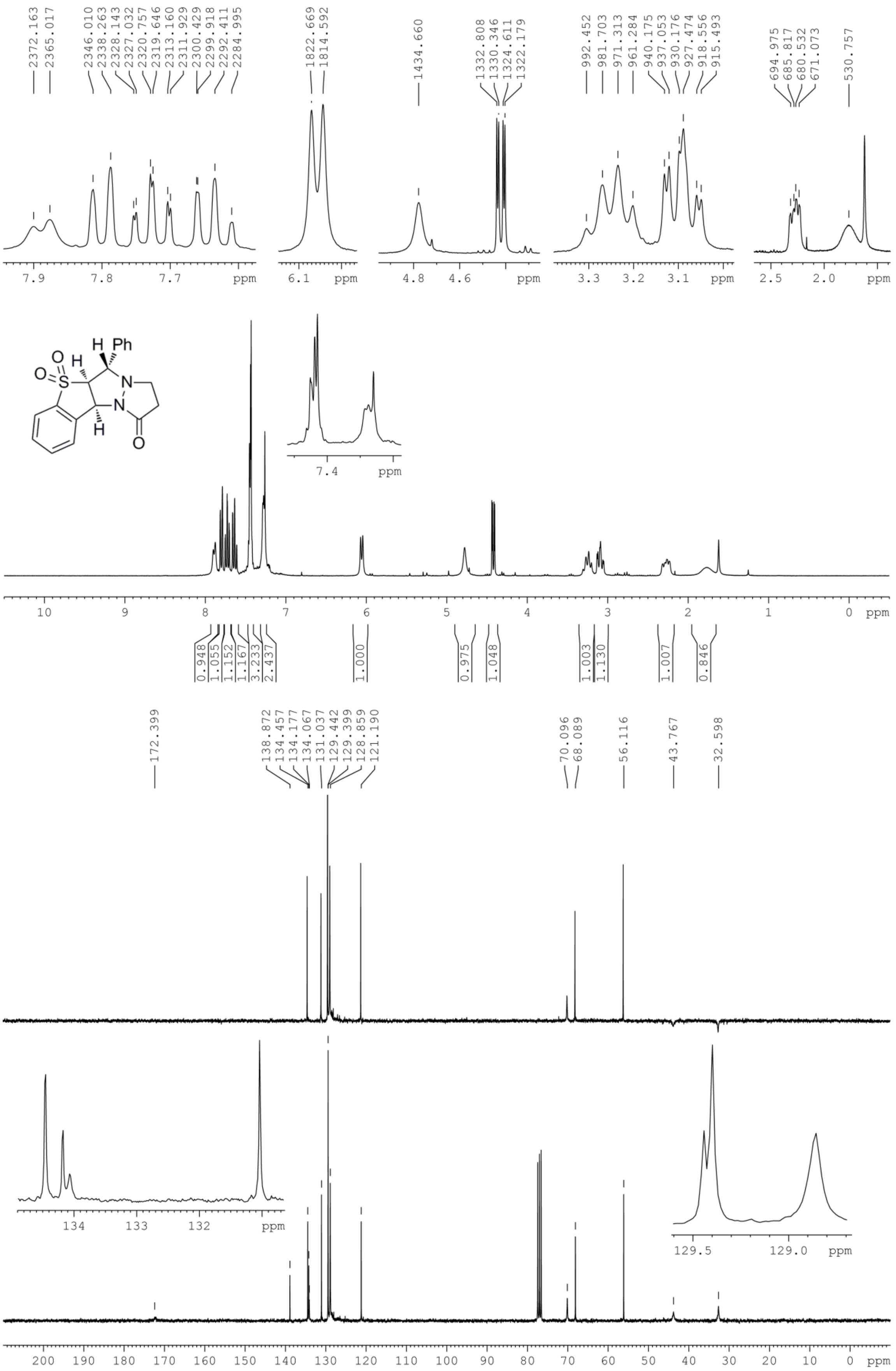

13. Geometry-optimized structure for **3a-endo**

Optimized energy: −1390.9235364 Hartree (DFT/B3LYP/6-31G*)

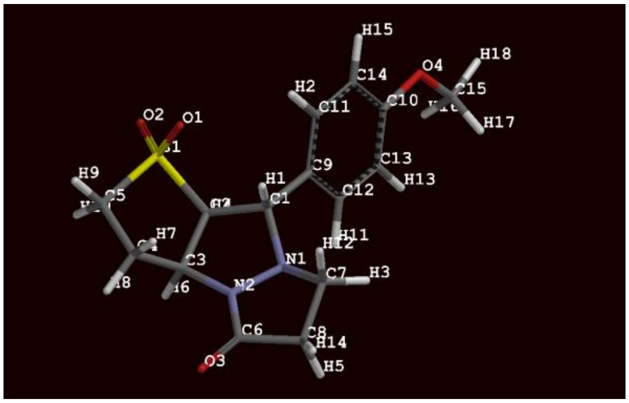

		Atom	X	Y	Z
1	S	S1	1.2909097	2.3699504	-0.0390958
2	C	C2	0.9527198	0.6874151	0.6565145
3	C	C3	2.3431975	0.0365402	0.9107221
4	C	C4	3.4554967	0.8682306	0.2203702
5	C	C5	3.0736445	2.3389358	0.3818677
6	H	H4	0.3840958	0.8474016	1.5735063
7	H	H6	2.5409681	-0.0237534	1.9885476
8	H	H7	3.4893978	0.6108523	-0.8423785
9	H	H8	4.4339969	0.6414497	0.6489249
10	H	H9	3.5898315	3.0195125	-0.2986168
11	H	H10	3.1684460	2.6986623	1.4110559
12	O	O1	1.1720143	2.3081096	-1.5090455
13	O	O2	0.5726042	3.3963217	0.7285479
14	C	C1	0.2150632	-0.2720755	-0.3175187
15	H	H1	0.5430366	-0.0259222	-1.3403634
16	N	N1	0.7689126	-1.5743868	0.1058846
17	N	N2	2.1624652	-1.2768370	0.3371450
18	C	C6	3.0106334	-2.3034300	0.0686703
19	O	O3	4.2159993	-2.3140000	0.2754890
20	C	C7	0.8367022	-2.6485663	-0.9085625
21	H	H3	-0.0736086	-3.2502893	-0.8732843
22	H	H12	0.9452222	-2.2221525	-1.9199916
23	C	C8	2.1153774	-3.4076314	-0.5146735
24	H	H5	1.9225780	-4.1478772	0.2715963
25	H	H14	2.6060922	-3.9143885	-1.3482398
26	C	C9	-1.2942663	-0.2231651	-0.2494660
27	C	C10	-4.1010189	-0.0563076	-0.1197201
28	C	C11	-2.0049250	0.6080711	-1.1296254
29	C	C12	-2.0113292	-0.9629918	0.6933613
30	C	C13	-3.4048362	-0.8910900	0.7625230
31	C	C14	-3.3888136	0.6961581	-1.0657817
32	H	H2	-1.4634000	1.2009161	-1.8626954
33	H	H11	-1.4741679	-1.6152055	1.3759320
34	H	H13	-3.9293849	-1.4861871	1.5014928
35	H	H15	-3.9426716	1.3397375	-1.7419785
36	O	O4	-5.4565045	0.0913840	-0.1441716
37	C	C15	-6.2304940	-0.6404353	0.7929250
38	H	H16	-5.9776281	-0.3677844	1.8260086
39	H	H17	-6.1025576	-1.7235335	0.6643889
40	H	H18	-7.2702623	-0.3745473	0.5942245

14. Geometry-optimized structure for **3a-**exo

Optimized energy: −1390.9250155 Hartree (DFT/B3LYP/6-31G*)

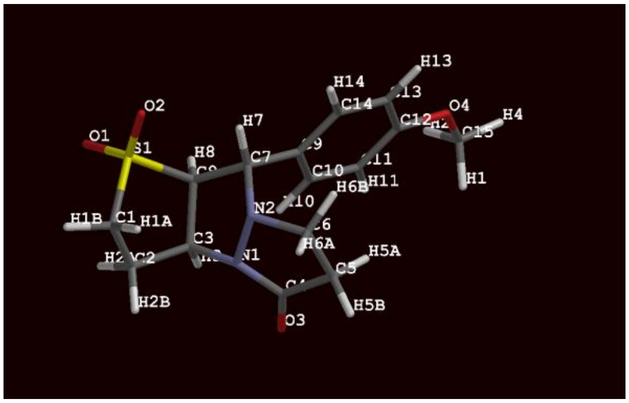

		Atom	X	Y	Z
1	S	S1	3.0175787	-1.4200341	-0.1323919
2	C	C1	3.9257867	0.1562811	-0.0314463
3	H	H1A	3.7694951	0.5526280	0.9743508
4	H	H1B	4.9829544	-0.0540021	-0.2069815
5	N	N1	1.1275775	1.6248402	0.0560710
6	O	O1	3.4627910	-2.1308093	-1.3450182
7	O	O2	2.9714260	-2.1124958	1.1653697
8	N	N2	0.9480530	0.8102202	1.2300002
9	C	C2	3.2565377	1.0026730	-1.1134665
10	H	H2A	3.5882031	0.6652189	-2.1012300
11	H	H2B	3.5063696	2.0620113	-1.0120649
12	O	O3	-0.0901064	3.1354751	-1.1953916
13	C	C3	1.7215128	0.8178558	-1.0003442
14	H	H3	1.2385264	1.0961328	-1.9407940
15	C	C4	0.0927752	2.5231196	-0.1578945
16	C	C6	0.0384492	1.6155089	2.0820243
17	H	H6A	0.6585843	2.1634843	2.7983984
18	H	H6B	-0.6253312	0.9529610	2.6394377
19	C	C5	-0.7059134	2.5858295	1.1433102
20	H	H5A	-1.7338069	2.2828541	0.9221358
21	H	H5B	-0.7351370	3.6105501	1.5244999
22	C	C8	1.3814921	-0.6415561	-0.5453918
23	H	H8	0.9660792	-1.2689719	-1.3344351
24	C	C7	0.4669653	-0.5131424	0.6857881
25	H	H7	0.7393852	-1.2672383	1.4300138
26	C	C9	-1.0165126	-0.6389933	0.3836062
27	C	C10	-1.6155466	-0.1831584	-0.7970831
28	H	H10	-1.0168247	0.2568235	-1.5893061
29	C	C11	-2.9950018	-0.2705952	-1.0016482
30	H	H11	-3.4147318	0.0969812	-1.9309079
31	C	C12	-3.8112507	-0.8304901	-0.0118841
32	C	C13	-3.2273644	-1.3085406	1.1712772
33	H	H13	-3.8685194	-1.7571812	1.9235579
34	C	C14	-1.8555309	-1.2133344	1.3559925
35	H	H14	-1.4182613	-1.6035036	2.2726837
36	O	O4	-5.1629423	-0.9628245	-0.1023531
37	C	C15	-5.8084939	-0.4997395	-1.2802691
38	H	H2	-5.4547200	-1.0363310	-2.1701335
39	H	H1	-5.6584145	0.5781561	-1.4237979
40	H	H4	-6.8712965	-0.7005212	-1.1359348
